# Effects of Dietary Rosemary Extract Supplementation on Pork Quality of Chato Murciano Breed during Storage

**DOI:** 10.3390/ani11082295

**Published:** 2021-08-04

**Authors:** Irene Peñaranda, Sonia Mariella Auqui, Macarena Egea, María Belén Linares, María Dolores Garrido

**Affiliations:** Department of Food Science and Technology, Veterinary Faculty, University of Murcia, 30071 Murcia, Espinardo, Spain; irene.penaranda@um.es (I.P.); soniamariella.auqui@gmail.com (S.M.A.); macarena.egea@um.es (M.E.); blinares@um.es (M.B.L.)

**Keywords:** pig, rustic, Chato Murciano, rosemary, meat quality

## Abstract

**Simple Summary:**

Chato Murciano pigs are a protected breed of great interest from a health point of view since their meat has a high content of polyunsaturated fatty acids (PUFA). However, this could lead to technological problems that could negatively affect the quality and shelf life of this meat. Therefore, the dietary supplementation with Rosmarinus officinalis L. extract could have an impact on the antioxidant and antimicrobial properties of the Chato Murciano pork and thus control its deterioration. In the present study, rosemary extract was not effective in improving the pork quality packaged under modified atmosphere over storage time, but it delayed microbial growth without affecting the sensory profile. Thus, rosemary extract could be used to enhance the shelf-life of meat by controlling microbial spoilage.

**Abstract:**

(1) Background: The effect of *Rosmarinus officinalis* L. dietary supplementation on the pork meat quality of the Chato Murciano breed of pigs was evaluated during 21 days of storage. (2) Methods: Twenty-one castrated male pigs were divided into two groups. One group was fed a control diet (group C), and the other group consumed the same diet plus a 1000 ppm supplement of deodorized rosemary extract (group R). (3) Results: While the inclusion of rosemary extract in the pig diet did not produce notable changes in the technological parameters analysed in the meat, the lower microbiological count obtained in meat pointed to the antimicrobial effect of the extract. The storage time had a significant effect on all the parameters studied in both groups (C and R). Thus, lipid oxidation increased and the colour of the meat deteriorated, at the same time as the microbial counts and the deterioration of the sensory attributes increased. (4) Conclusions: Therefore, a certain antimicrobial effect of rosemary was observed in the meat of Chato Murciano.

## 1. Introduction

The rustic pig breed “Chato Murciano” from south-eastern Spain (Murcia Region) is classified by the Spanish Ministry of Agriculture, Fishing and Food as a breed in need of special protection and in danger of extinction (B.O.E 21/11/97). Fortunately, national and international bodies (the European Union, FAO (Rome, Italy), etc.) continue to support Conservation and Recovery programmes to help maintain global biodiversity through such means as the EU Biodiversity Strategy to 2020. In the mid-20th century, Chato Murciano pig production was replaced by the arrival of commercial cross-breeds and many of the traditional meat products produced from this rustic breed were no longer manufactured [1]. The pig meat of the Chato Murciano breed has a high intramuscular fat content (approximately 7.9%) [1] with a high percentage of polyunsaturated fatty acids (AGPI). Whilst this may be of interest from a health point of view, it can lead to technological problems, including very long curing times, a soft consistency, and lipid oxidation, among others, which negatively affect the quality and shelf life of the meat and meat products [2].

In addition to oxidative processes, quality can be affected by microbial growth and hence sensory changes, resulting in unpleasant odours and changes in meat colour, flavour, and texture. Strategies to control such deterioration include the use of antioxidants, antimicrobials, and different preservation and/or packaging systems [3]. However, due to concerns regarding the safety and toxicity of synthetic antioxidants, research is increasingly focusing on natural antioxidants derived from plant sources to help preserve the sensory quality of meat, since consumer acceptance of natural antioxidants is greater than that for synthetic antioxidants [4].

Various herbs and plant extracts, mainly from the Labiatae family such as rosemary, thyme, oregano, and sage, have been extensively studied for their antioxidant and antimicrobial properties, especially for the use as natural animal feed additives [3,5]. However, to date, rosemary extract is the only one to which an E code (E-392) has been assigned, in accordance with EU regulations [6]. *Rosmarinus officinalis* L., a Mediterranean plant rich in phenolic compounds, is recognized for its potent antioxidant activity, as well as therapeutic, anticancerogenic, and antiviral properties [7]. Most constituents of rosemary are phenolic diterpenes that are considered natural-functional ingredients [8]. Moreover, the main phenolic compound is carnosic acid, whose antioxidant activity is approximately seven times higher than that of synthetic antioxidants [9]. However, the use of rosemary as an additive is often limited due to its characteristic odour, which makes it unsuitable in certain foods (e.g., meat and fish). Therefore, the aim of the research described herein was to study the antioxidant and antimicrobial effect of deodorized rosemary extract in the diet, on the quality, and the shelf life of fresh meat from the Chato Murciano pig breed packaged in modified atmosphere and stored refrigerated for 21 days.

## 2. Materials and Methods

### 2.1. Animals

The experimental procedures used in this study were in compliance with the Directive 2010/63/EU and Royal Decree 53/2013 laying down basic rules for the care and handling of research animals [10]. Twenty-one castrated male Chato Murciano pigs were randomly selected from a commercial population belonging to the Integrated Center of Formation and Agrarian Experiences located in Lorca (CIFEA, Murcia, Spain), and randomly assigned to two treatment groups according to the feeding system. During the fattening stage, the Control group (C, *n* = 10) was fed exclusively with commercial feed (S.A.T La Rambla N° 1.932, Totana, Murcia, Spain, Table 1), while the animals in the Rosemary extract group (R, *n* = 11) were fed the same feed but containing 1000 ppm of deodorized rosemary extract (Ingrenat S.L., Santa Ana, Murcia, Spain) according to existing literature [3,9].

The animals within each group were reared under a semi-intensive system (CIFEA, Murcia, Spain), each group separated according to the feed they received, which was provided ad libitum, where they enjoyed several hours a day of outdoor breeding where their food supply was obtained directly from the plants and trees where they lived (figs, peaches, apricots, etc.). Animals were off feed with access to water, for 12 h prior to slaughter and were transported according to Directive 86/609/EEC, modified by 2003/63/EC and law 32/2007 regarding vaccination and deparasitation recommended by the Health Authority. The pigs (153.00 ± 11.98 kg live weight) were gas-stunned and slaughtered in the “La Comarca” slaughterhouse (Lorca, Spain), exsanguinated and eviscerated in accordance with Council Regulation (EC) No 1099/2009 [11], following the criteria of regional meat market demand typical of this breed [1].

### 2.2. Sample Preparation

The carcasses, with an average hot weight of 123.4 ± 3.02 kg and a yield of 80%, were chilled at −9 °C for two hours and then kept at 4 °C. Two hours post-mortem, the *longissimus thoracis et lumborum* muscle was obtained and transported under refrigeration to the Food Technology Department of Murcia University (Spain), where the muscle was stored (24 h post-mortem) until being processed. The meat pieces from both treatments (C and R) were cut into 1.5 cm fillets, packaged in a modified atmosphere, and placed in polystyrene trays B5-37 (Aerpack), which were placed in low gas permeability BB325 bags (Cryovac). The air in the trays was evacuated and replaced by the gas mixture EAP20 (Carburos Metálicos, S.L., Barcelona, Spain) composed of 70% O_2_ and 30% CO_2_, with commercial R-RE packaging (Industrias RAELMA, S.L., Madrid, Spain). The atmosphere of the containers was replaced following a double vacuum sequence (760 mm Hg) and later filled with the EAP20 mixture. The final composition of the gases was checked with a Pack 12P gas analyzer (Abiss). After packaging, the samples were stored under refrigeration (4 °C) in a display case (Helkama, Finland) illuminated with fluorescent light (620 lux) simulating the usual conditions of commercialization. The control analyses were performed on days 0, 7, 14, and 21.

### 2.3. Meat Quality Measurements

The proximate composition was assessed by applying the following ISO norms: moisture, ISO 1442:1997 [12]; total ash ISO 936:1998 [13]; total protein ISO 937:1981 [14] (Kjeldahl N × 6.25); intramuscular fat content, ISO 1443:1979 [15], using petroleum ether (40–60 °C) as the solvent in a Soxhlet. The water-holding capacity (WHC) was also calculated [16].

L*, a*, b* colour parameters were measured using a CR-400 Chroma Meter (Minolta Ltd., Milton Keynes, United Kingdom) calibrated against a standard white tile (8 mm diameter aperture, d/0 illumination system, D65 illuminant, and a 2° standard observer angle) on the sample surface from three randomly chosen spots. The parameters referred to *Chroma* (C *) and *Hue angle* (H°) were obtained by using the following equations [7]:

$$Chroma={\left(a^{*2}+\right)}^{1/2},$$

$$Hue\;angle=tan^{-1}\left(b^{*}/a^{*}\right).$$

The pH (24 h after slaughter) was measured with a pH meter (Crison GLP21, Eutech, Singapore) equipped with a penetrating glass electrode [16].

Lipid oxidation was quantified by the thiobarbituric acid reactive substances (TBARs) method, as described by Botsoglou et al. [17]. The absorbance was measured at 532 nm in a spectrophotometer (Pye Unicam Ltd., Cambridge, UK) and the results are expressed as mg malondialdehyde (MDA)/kg^−1^ of meat. All the chemicals were provided by Sigma-Aldrich (St. Louis, MO, USA). All determinations were performed in triplicate for each meat sample.

### 2.4. Microbiological Analysis

To evaluate the microbiological quality, a 10 g sample of muscle was mixed with 90 mL buffered peptone water 0.1% *w*/*w* (Microkit BCD046, Madrid, Spain) in a stomacher (IUL Instruments, GmBH, Köningswinter, Germany), inside an aseptic cabinet (Telstar, Bio–II–A. Tarrasa, Spain) and serial dilutions were prepared. Bacterial counts were determined as follows: Total Aerobic were determined by the pour plate method in Plate Count Agar (Oxoid CM0325, Basingstoke, UK) incubated at 30 °C for 42 days [18]; Psychrophilic bacteria were analysed in Plate Count Agar (Oxoid CM0325, Basingstoke, UK) and incubated at 4 °C for 10 days [19]; Lactic Acid Bacteria were counted on Man Rogosa and Sharpe Agar (Oxoid CM0361, Basingstoke, UK) (30 °C for 2 days) [20]^;^ violet red bile glucose agar (Oxoid CM485, Basingstoke, UK) was used for *Enterobacteriaceae* incubated at 37 °C for 24 h [21]; Total Coliforms were determined using a violet red bile glucose agar (Oxoid CM017, Basingstoke, UK) after incubation at 37 °C for 24 h, *Pseudomonas* were determined by plate seeding on agar base (Oxoid, Basingstoke, UK) (28 °C-24 h), and Moulds and Yeasts was enumerated with Rose Bengal Agar + Chloramphenicol (Oxoid CM0549, Basingstoke, UK) (25 °C for 5 days) [22]. Plating was performed in duplicate, and the results were expressed as log colony-forming unit (CFU) g^−1^.

### 2.5. Sensory Analysis

Eight panellists chosen from the Department staff of Food Technology of the University of Murcia, all experienced in the profile assessment of different meat products, were selected and trained according to ISO 8586:2012 [23]. Six theoretical-practical sessions of 1.5 h were held for specific training on each of the products (fresh meat and cooked meat). To assess the effect of rosemary extract on meat quality and shelf life, a Quantitate Descriptive Analysis (QDA) test was performed for both products, using a structured 7-point scale (0 = not perceptible; 7 = maximum perceptibility). The attributes evaluated were meat odour, rancid odour, acid odour, meat colour, fat colour, meat flavour, rancid flavour, hardness, and juiciness. Fresh meat samples were only analyzed for appearance and aroma attributes and cooked samples included texture and flavour attributes. The sensory evaluation of the meat was carried out according to ISO 4121:2003 [24] in a standardized room of the Department of Food Technology of the University of Murcia. The analyses were carried out in the morning at 10:00 h and in each session a total of six samples were analysed by each panellist.

The sensory analysis of fresh and cooked meat is outlined below:

The refrigerated pork fillets were tempered at room temperature 10 min before the sensory analysis and the QDA tests were performed on days 0, 7, 14 and 21. Each panellist analysed a total of three samples per treatment (including three replicates of the control and three replicates of the rosemary extract group) and storage day, in four total sessions.

To simplify the sensory analysis of cooked meat, samples were considered only at days 0 and 14 of storage, these two times being chosen as extremes, since on day 21 (the last day of the trial) the microbiological counts were higher than that established by the Commission Regulation EC 2073/2005 [25]. The fillets were cooked on a double-sided grill (Media Liscia; Silanos Lavastoviglie Industriali, Pioltello, Italy) previously heated at 180 °C for 10 min. Both the top and the bottom of the grill were covered with aluminium foil and the fillets were cooked for 4 min until reaching an internal temperature of 72 °C (T200 portable thermometer, Digitron Instrumentation Ltd., Merd Lane, Hertford, United Kingdom). The cooked samples were trimmed of any external connective tissue, cut into 1.5 cm × 2 cm pieces and then wrapped in aluminium foil coded with a three-digit random number and kept in a sand bath at 60 °C until tasting [26]. Sample presentation was balanced to account for order and carryover effects [27]. Three meat samples per treatment (control and rosemary extract) and storage days (0 and 14) were evaluated by each panellist, over a total of two sessions. Mineral water and unsalted bread were provided for mouth rinsing between samples.

### 2.6. Statistical Analysis

All data were analysed with the statistical package SPSS 24 software package (SPSS, Chicago, IL, USA). The performance parameters were analysed by one-way analysis of variance (ANOVA) to evaluate the effect of dietary treatment with deodorized rosemary extract. The pH, colour (L*, a*, b*, Chroma and Hue angle), oxidation (TBARs) and microbiological data were analysed separately through a two-way analysis of variance (ANOVA), considering the effects of dietary treatment (C and R) and storage time (Time; days 0, 7, 14, and 21) as fixed sources of variation and the individual pork as a random effect. For the sensorial data, a two-way ANOVA was performed, considering the effects of dietary treatment (C and R) and storage time (time; fresh meat: 0, 7, 14 and 21 days, cooked meat: 0 and 14 days) as fixed sources of variation and the individual pork and panellists as a random effect. Comparisons between means were performed using a Tukey test and differences were considered significant at the *p* < 0.05 level.

## 3. Results and Discussion

### 3.1. Meat Quality

The results for the proximal composition are presented in Table 2. As can be seen, there were significant differences (*p* < 0.05) in the moisture content, fat content, and WHC between the two groups (C and R). The C samples showed higher fat values, which would be related to the lower moisture content of this batch, while the opposite effect was observed in the R samples. In addition, R samples had lower WHC values. However, there were no statistical differences (*p* ≥ 0.05) in terms of the protein and ash contents. In general, there was no clearly significant effect of rosemary extract on the parameters evaluated. These results show a behaviour similar to that observed in other studies [28,29], where no changes were found in the proximal composition of Chato Murciano or Iberian pork in samples from animals fed rosemary.

Table 3 shows the effect of feeding and storage time on the quality parameters of meat. No significant differences in the pH (*p* ≥ 0.05) between the C and R groups were observed, since the average pH value in both groups remained constant throughout storage (pH 5.17–5.38). Similar results were observed in Chato Murciano [1] and castrated Iberian pork [29], and in studies conducted with different doses of rosemary extract, rosemary or oregano essential oil, vitamin E, tea catechins, among others, added to the feed of pigs, lambs, and chickens [3,5,30], which indicates that the pH of the sample is not affected by the incorporation of certain extracts in the animal diet.

The lipid oxidation (TBARs values) in both treatments (C and R) increased significantly (*p* < 0.05) over the storage time, the highest values being obtained at day 21 in both groups (0.55 and 0.47 mg MDA/kg meat), respectively. Therefore, in the TBARs values, lower values were observed in pork from animals fed the rosemary diet compared with the control animals. Gray et al. [31] suggested a threshold value of 1 mg MDA/kg meat before rancidity could be detected, and our values were below that threshold. In Iberian pork stored for 10 days in refrigeration, Ramírez and Cava [32] obtained results similar to those of our study (day 10: 0.141 mg MDA/kg *Longissimus thoracis*). Neither did the diet (*p* ≥ 0.05) affect lipid oxidation, as measured in the meat over 21 days of refrigerated storage, indicating a lack of effect of the rosemary extract used, perhaps because the concentration added was not high enough to improve the shelf life of the meat. These results agree with those of Liotta et al. [7] in pigs fed a diet supplemented with rosemary extract, suggesting that there was a significant loss of phenolic compounds as lipid oxidation progressed since the extract would be eliminated in the urine or perhaps it biotransformed into forms not available within the digestive system. Although there are numerous studies on the antioxidant activity of rosemary, in which such activity was attributed to various polyphenolic compounds (carnosic acid, carnosol, rosmarinic acid, rosmanol, and ursolic acid) which accumulate in the lipid membranes of the cells and contribute to retarding the autoxidation rate [8,9,28].

During the storage time, L* values increased in both C and R samples (*p* < 0.05), especially from day 7 onwards, although the increase was not so pronounced in the group fed rosemary, which may have been due to an antioxidant effect that helped the colour preservation. Similar results were obtained by Liotta et al. [7] for the L* coordinate and the effect of storage time (5 days) in pigs fed rosemary extract (0.1%). In the case of the a* coordinate, its value decreased during storage for both treatments in our study (*p* < 0.05), which indicated a loss of redness in the fresh pork. Regarding the average Chroma values, the R meat samples showed greater colour stability than the control sample. However, for both the a* coordinate and Chroma, a significant increase was observed to have occurred by day 7 in both groups, which could be due to a loss of mitochondrial respiration during storage, which would increase the oxygen on the surface of the muscle [33]. Similar results were obtained by Ramirez and Cava [32] in Iberian pigs during refrigerated storage. As regards the diet, the a* coordinate and Chroma were affected (*p* < 0.05) by the incorporation of rosemary extract in the animal feed, as seen on days 0, 7, and 14 of storage, although the values were lower in the R group [7]. The values of b* and Hue angle varied between −1.02 and 3.79 (b*) and −5.05 and 17.75 (H°) during days 0 and 21 respectively. It showed a highly significant increase (*p* < 0.05) between days 0 and 7 of storage, which was more pronounced in the C samples, which meant an increase in the yellowish tones that some consumers might find unattractive [32]. Our values were similar to those observed by other authors in Chato Murciano pigs semi-intensively reared (b*: −1.09) [16] and raised outdoors (b*: 1.4) [1], and in Chato Murciano pigs crossed with Large White (b*: −1.01) [34]. However, both parameters were much lower than those found for Iberian pigs (b*: 5.16, H°: 27.2) [29], Siciliano pigs fed a 0.01% rosemary extract supplement (b*: 15.57, H°: 0.86) [7], and for pigs with a dietary supplement of 0.05% rosemary essential oil 0.05% (H°: 23.08) [35], as the meat colour is influenced by animal breed, age, sex, and feed [34]. In general, the values of b* and Hue angle were not greatly affected by the incorporation of rosemary extract in the animal feed. Several authors have described the same behaviour in animals given feed supplemented with different doses of rosemary extract [5,7].

### 3.2. Microbiological Analysis

The results of the microbiological analysis of pork packed in a modified atmosphere and stored under refrigeration for 21 days are presented in Table 4. Microbiological counts of Mesophiles, Psychrophiles, *Enterobacteriaceae*, Coliforms, *Pseudomonas* spp., Lactic Bacteria, Molds and Yeasts increased significantly (*p* < 0.05) over the 21 days of storage in both groups (C and R). The Total Mesophilic count on days 0 and 7 were within the legally permitted limit of 6 CFU/g fresh meat [25] but at day 14, the values exceed this limit in both batches. Some authors have shown that the microbial deterioration generated by mesophiles occurs when they reach 7–8 CFU/g of fresh meat [36], making the product unfit for consumption. After 21 days of storage, the *Enterobacteriaceae* count was also above the legal limits (10^2^ CFU/g fresh meat) [25].

Regarding the effect of supplementing feed with rosemary extract, the growth of mesophiles, moulds and yeasts was more pronounced (*p* < 0.05) in R than in C samples on all sampling days during storage, which suggests an antimicrobial effect of rosemary extract on the quality of fresh meat [3]. In the psychrophilic and lactic bacteria counts significant differences (*p* < 0.05) were only observed between the feeding groups on day 21 of storage, when the R samples had a lower microbiological count than C samples. Similar results were obtained by Morán et al. [5] and Nikmaram et al. [3] in animals given feed supplemented with rosemary (turkey), carnosic acid (lamb), or rosemary extract (shrimp). The antimicrobial effect of rosemary on psychrophilic bacteria seems to be less intense than it is on other microorganisms because these bacteria can still proliferate under refrigerated conditions (since they can grow at temperatures lower than 7 °C) [3].

Significant differences (*p* < 0.05) between dietary treatments (C and R) as regard the total number of *Coliforms and* *Pseudomonas* were only found on day 7 for coliform bacteria and on days 0 and 7 of storage for *Pseudomonas*, when rosemary-fed animals (R) had the lower levels of these bacteria. It is possible that the incorporation of rosemary contributed to inhibiting the growth of coliforms and *Pseudomonas* in the samples [37]. Besides, *Pseudomonas* is among the most sensitive species to CO_2_, so the use of modified atmosphere could have inhibited growth, thus facilitating the possible effect of carnosic acid [5]. Conversely, in the case of *Enterobacteriaceae* counts, significant differences between treatments (*p* < 0.05) were observed at any time, giving the R samples the slightly lower values. A similar situation during storage was described by Lauzurica et al. [36] and Morán et al. [5], although their counts differed.

There are several studies in which the inhibitory effect of plant extracts, including rosemary essential oil or extracts on microbial and fungal development has been demonstrated [37]. Generally, the inhibitory effect of the extracts was attributed to their polyphenolic composition, since the action mechanism of low molecular weight phenolic acids involves disturbing the cytoplasmic membrane as the non-dissociated acid is diffused across the membrane, causing acidification of the cytoplasm and sometimes cell death. However, it should be noted that the antimicrobial activity is influenced by various factors, including pH, type of microorganism, food components, temperature, and properties of the natural components of the extracts used [3].

### 3.3. Sensory Analysis

The results of sensory analysis in fresh and cooked meat of Chato Murciano pig stored under controlled conditions are presented in Table 5 (fresh meat) and Table 6 (cooked meat).

#### 3.3.1. Fresh Meat

The sensory attributes of meat odour, meat colour, and fat colour, all related to the quality of fresh meat, decreased significantly (*p* < 0.05) during the storage time for both treatment groups (C and R), presenting maximum values at day 0 (7.0 in both C and R) and minimum values at day 2, when the following values were recorded: meat odour (4.23 in R and 3.0 in C), meat colour (4. 40 in R and 4.07 in C), and fat colour (4.95 in R and 4.50 in C). By contrast, the attributes rancid odour and acid odour, which are related to the deterioration of meat significantly increased (*p* < 0.05) with storage time, the highest values being recorded on day 21. The maximum score of 2.11 for rancid odour on day 21 in the C group, while having a very low perception score, could have been related to the low rates of TBAR_S_ found, since they remained below the perception threshold (1 mg MDA/kg) throughout storage [31]. Gorelik and Kanner [38] indicated that lipid peroxidation promotes the formation of rancid and other unpleasant odours, the oxidation of myoglobin resulting in deterioration in the colour of fresh meat. Regarding animal feed, rosemary did not significantly affect the evaluated attributes (*p* ≥ 0.05), except on days 7 and 21 of storage in the case of meat odour and on day 21 for meat colour and fat colour, when the R samples presented the highest values for these attributes, this confirms the positive effect of the rosemary extract on the odour and colour of the meat [39]. Additionally, on days 7 and 14 of storage, the pigs fed with rosemary showed the lowest rancid odour value. Dietary supplementation with rosemary has been shown to reduce rancid odour in the meat after 7 days of storage due to rosemary diterpenes (carnosic acid, rosmarinic acid, carnosol, rosmanol, epirosmanol, and isorosmanol) which act as preservatives against oxidative processes, Nieto et al. [40] obtained rancid odour scores similar to those in this research in lambs fed a supplement of 10–20% rosemary leaves (C: 1.80, R10%: 1.37, R20%: 1.14 at day 7 of storage). In general, the changes produced in fresh meat did not lead to large differences in colour or odour, reflecting the results shown for the colour coordinates.

#### 3.3.2. Cooked Meat

For the sensory analysis of cooked pork, both the C and R samples were analyzed on days 0 and 14 of storage, since on day 21 the samples exceeded the maximum permitted microbial load. As expected, the scores for meat odour, meat colour and meat flavour, hardness and juiciness decreased with the storage time, while the attributes linked to deterioration of the product, rancid odour, acid odour and rancid flavour increased. Therefore, in terms of the storage time, some of the evaluated attributes from both treatments were significantly affected (*p* < 0.05), except meat odour, acid odour, hardness and juiciness in the R samples and meat colour in the C samples. These results are similar to those obtained by Echegaray et al. [41] in Celta pig breed, since the values obtained for the sensory attributes of the meat (meat colour, surface discolouration, and fresh odour) fell during refrigerated storage, both in the pigs fed a commercial feed and in those fed supplemented with chestnuts.

Neither meat odour nor meat colour showed a clear trend regarding the effect of storage time in either treatment group. The attribute meat flavour decreased slightly during the storage time in both groups, accompanied by an increase in rancid flavour attribute. Regarding the effect of the diet, no significant differences (*p* ≥ 0.05) were observed between the control animals and those fed a rosemary extract supplement, except on day 0 for the texture attributes (greater hardness and juiciness and a lower meat flavour in the R samples), as cooking has been shown to limit the antioxidant activity of the endogenous polyphenols in rosemary [40]. There are not many bibliographical references to the effect of dietary herbs and spices on the sensory quality of cooked pork. Janz et al. [34] found no differences in the flavour of meat from pigs fed with 0.05% of essential oil/rosemary oleoresins compared with the control. Lara et al. [42] reported no significant differences in sensory attributes between pork patties treated with 0.03% of rosemary and 0.1% of lemon balm and packaged in a protective atmosphere (70% N_2_ + 30% CO_2_) and control samples. Similar results are mentioned by other authors, including Morán et al. [5], who found no differences in the sensory quality of meat from control lambs or those fed a carnosic acid and vitamin E supplement. However, Smeti et al. [30] noted that oral administration of 0.06% rosemary essential oil improved the flavour (C: 6.25, R: 7.33) and juiciness (C: 6.13, R: 7.16) of lamb meat, finding that the dose and also the application format are relevant for the sensory quality. In this study, the addition of rosemary extract to the diet did not improve meat flavour (C: 6.47, R: 6.25) but did improve juiciness at day 0 of storage with scores of 4.05 (R) and 3.74 (C). This could be due to the protection exerted by the rosemary extract against oxidation of endogenous proteases during the ageing process, thus reducing the amount of water expelled from the intra- and extramyofibrillar spaces of the cells [5].

## 4. Conclusions

The incorporation of 1000 ppm of deodorized rosemary extract in the feed of Chato Murciano pigs did not affect the physical-chemical parameters of fresh meat or the sensory parameters of fresh and cooked meat. However, a certain antimicrobial effect was found for rosemary in Chato Murciano meat, and it especially appears to have some inhibitory effect on psychrophilic and lactic acid bacteria when the meat is subjected to long periods of conservation (21 days). In general, a significant effect of storage time on the physical-chemical, microbiological, and sensory parameters were observed in both the control group and those fed the rosemary extract.

## Figures and Tables

**Table 1:** Ingredients and chemical composition of the commercial feed of Chato Murciano.

Ingredients (%)	
Barley	47.00
Wheat	33.50
Sunflower seed meal	6.00
Soy meal	6.00
Corn gluten feed	5.00
Calcium carbonate	1.56
Butter	0.50
Calcium phosphate	0.20
Sodium chloride	0.40
Protein	13.30
Fat	2.20
Cellulose	5.70
Ash	5.10
Vitamin A	15.000 U.I./kg
Vitamin D3	1500 U.I.
Vitamin E	20 mg/kg
Copper	20 mg/kg
(E-1614) 6-phytase	500 FYT/kg

Proximal composition: dry matter 89%.

**Table 2:** Proximal composition of Chato Murciano pork (*Longissimus thoracis et lumborum*, mean ± SD).

Parameters	C		R		*p*-Value
	Mean	SD	Mean	SD	
N	11		10		
Moisture (%)	68.40	2.64	70.97	1.11	*
Total ash (%)	1.08	0.07	1.05	0.26	NS
Total protein (%)	23.84	1.79	23.56	0.88	NS
Total fat (%)	7.98	2.24	5.43	1.58	**
WHC (%)	90.89	2.19	87.53	1.42	**

Parameters WHC: water holding capacity. Dietary treatments, C: commercial feed, R: commercial feed supplemented with 1000 ppm of deodorized rosemary extract. The *p* values indicated by * and ** for *p* < 0.05 and *p* < 0.01, respectively. NS: not significant *p* ≥ 0.05.

**Table 3:** Effect of feeding and storage time on the quality parameters of Chato Murciano pork (*Longissimus thoracis et lumborum*) stored under controlled conditions (mean ± SD).

Parameters	Diet/Storage	Day 0	Day 7	Day 14	Day 21	*p*-Value
pH	C	5.30 ± 0.17	5.32 ± 0.21	5.30 ± 0.05	5.38 ± 0.16	NS
	E	5.17 ± 0.15	5.2 0.21	5.25 ± 0.31	5.20 ± 0.17	NS
		NS	NS	NS	NS	
TBARS	C	0.04 ± 0.01 ^a^	0.07 ± 0.03 ^ab^	0.16 ± 0.04 ^b^	0.55 ± 0.21 ^c^	***
	E	0.05 ± 0.01 ^a^	0.08 ± 0.02 ^a^	0.14 ± 0.04 ^b^	0.47 ± 0.07 ^c^	***
		NS	NS	NS	NS	
L*	C	44.88 ± 3.88 ^a^	49.82 ± 4.25 ^b^	50.35 ± 3.54 ^b^	50.72 ± 3.35 ^b^	**
	E	46.64 ± 3.93 ^a^	50.53 ± 4.41 ^ab^	51.82 ± 4.88 ^ab^	52.63 ± 4.01 ^b^	*
		NS	NS	NS	NS	
a*	C	13.12 ± 0.90 ^b,x^	15.68 ± 0.99 ^c,x^	14.14 ± 1.12 ^b,x^	11.69 ± 1.29 ^a^	***
	E	12.15 ± 0.85 ^a,y^	13.64 ± 0.78 ^b,y^	12.87 ± 1.59 ^ab,y^	11.95 ± 1.43 ^a^	*
		*	***	*	NS	
b*	C	−0.51 ± 1.23 ^a^	3.68 ± 1.09 ^b,x^	3.51 ± 1.21 ^b^	3.79 ± 1.37 ^b^	***
	E	−1.02 ± 1.51 ^a^	2.48 ± 1.26 ^b,y^	3.01 ± 1.21 ^b^	3.40 ± 1.16 ^b^	***
		NS	*	NS	NS	
C*	C	13.18 ± 0.87 ^ab,x^	16.09 ± 1.19 ^c,x^	14.56 ± 1.31 ^b,x^	12.29 ± 1.45 ^a^	***
	E	12.28 ± 0.88 ^a,y^	13.91 ± 0.83 ^b,y^	13.28 ± 1.50 ^ab,y^	12.45 ± 1.27 ^a^	**
		*	***	*	NS	
H°	C	−2.50 ± 5.42 ^a^	12.99 ± 3.12 ^b^	13.70 ± 4.02 ^b^	17.75 ± 5.49 ^b^	***
	E	−5.02 ± 6.75 ^a^	10.18 ± 5.15 ^b^	13.45 ± 5.60 ^b^	16.34 ± 5.86 ^b^	***
		NS	NS	NS	NS	

Results are given as the mean ± SD of three independent triplicates. Parameters TBARS was expressed as mg malondialdehyde/kg muscle, L*: Lightening, a*: red–green; b*: yellow–blue, C*: Chroma, H°: Hue angle. Diet C: commercial feed, R: commercial feed supplemented with 1000 ppm of deodorized rosemary extract. Values within a row (^a,b,c^ effect of storage time) and in column (^x,y^ effect of dietary treatment) with different superscripts differ significantly at *p* < 0.05. The *p* values indicated by *, ** and *** for *p* < 0.05, *p* < 0.01 and *p* < 0.001, respectively. NS: not significant *p* ≥ 0.05.

**Table 4:** Effect of feeding and storage time on the microbiological count (log ufc/g) of Chato Murciano pork (*Longissimus thoracis et lumborum*) stored under controlled conditions (mean ± SD).

Microorganisms	Diet/Storage	Day 0	Day 7	Day 14	Day 21	*p*-Value
Mesophiles	C	4.11 ± 0.31 ^a,y^	4.97 ± 0.46 ^b,y^	6.79 ± 0.30 ^c,y^	8.13 ± 0.38 ^d,y^	***
	E	3.43 ± 0.47 ^a,x^	4.16 ± 0.56 ^b,x^	6.38 ± 0.31 ^c,x^	7.80 ± 0.32 ^d,x^	***
		**	**	**	*	
Psychrophiles	C	3.07 ± 0.53 ^a^	4.03 ± 0.56 ^b^	6.69 ± 0.86 ^c^	8.36 ± 0.50 ^d,y^	***
	E	2.93 ± 0.30 ^a^	3.88 ± 0.37 ^b^	6.16 ± 0.65 ^c^	7.84 ± 0.56 ^d,x^	***
		NS	NS	NS	*	
*Enterobacteriaceae*	C	2.02 ± 0.38 ^a,y^	2.92 ± 0.30 ^b,y^	4.33 ± 0.49 ^c,y^	7.02 ± 0.48 ^d,y^	***
	E	1.84 ± 0.78 ^a,x^	2.37 ± 0.91 ^a,x^	4.22 ± 0.67 ^b,x^	6.64 ± 0.43 ^c,x^	***
		*	*	*	**	
Coliforms	C	1.75 ± 0.65 ^a^	2.48 ± 0.36 ^b,y^	3.60 ± 0.52 ^c^	6.44 ± 0.33 ^d^	***
	E	1.56 ± 0.45 ^a^	1.72 ± 0.75 ^a,x^	3.74 ± 0.42 ^b^	6.29 ± 0.51 ^c^	***
		NS	*	NS	NS	
*Pseudomonas*	C	2.79 ± 0.44 ^a,y^	3.61 ± 0.55 ^b,y^	3.80 ± 0.67 ^c^	7.64 ± 0.29 ^d^	***
	E	2.11 ± 0.36 ^a,x^	2.86 ± 0.45 ^b,x^	5.52 ± 0.32 ^c^	7.49 ± 0.18 ^d^	***
		**	**	NS	NS	
Lactic Acid	C	1.65 ± 0.41 ^a^	2.63 ± 0.42 ^b^	4.35 ± 0.60 ^c^	6.68 ± 0.32 ^d,y^	***
	E	1.41 ± 0.40 ^a^	2.44 ± 0.58 ^b^	4.11 ± 0.47 ^c^	6.19 ± 0.53 ^d,x^	***
		NS	NS	NS	*	
Moulds and Yeasts	C	1.48 ± 0.38 ^a,y^	2.52 ± 0.35 ^b,y^	3.81 ± 0.25 ^c,y^	5.42 ± 0.43 ^d,y^	***
	E	1.13 ± 0.19 ª^,x^	1.77 ± 0.72 ^b,x^	2.86 ± 0.46 ^c,x^	4.70 ± 0.47 ^d,x^	***
		*	**	***	**	

Diet C: commercial feed, R: commercial feed supplemented with 1000 ppm of deodorized rosemary extract. Values within a row (^a,b,c^ effect of storage time) and in column (^x,y^ effect of dietary treatment) with different superscripts differ significantly at *p* < 0.05. The *p* values indicated by *, ** and *** for *p* < 0.05, *p* < 0.01 and *p* < 0.001, respectively. NS: not significant *p* ≥ 0.05.

**Table 5:** Effect of feed and storage on the sensory attributes in fresh meat of Chato Murciano pork stored under controlled conditions.

Attributes	Diet/Storage	Day 0	Day 7	Day 14	Day 21	*p*-Value
Meat odour	C	7.00 ± 0.00 ^c^	5.93 ± 0.50 ^b,x^	4.55 ± 1.53 ^ab^	3.00 ± 1.45 ª^,x^	***
	E	7.00 ± 0.00 ^d^	6.25 ± 0.87 ^c,y^	4.60 ± 0.96 ^b^	4.23 ± 1.61 ª^,y^	***
		NS	*	NS	*	
Rancid odour	C	1.00 ± 0.00 ^a^	1.16 ± 0.37 ^a,y^	1.78 ± 0.83 ^ab,y^	2.11 ± 1.10 ^b^	***
	E	1.00 ± 0.00 ^a^	1.00 ± 0.00 ^a,x^	1.32 ± 0.47 ^a,x^	2.05 ± 0.64 ^b^	***
		NS	**	**	NS	
Acid odour	C	1.00 ± 0.00 ^a^	1.14 ± 0.41 ^a^	1.38 ± 0.54 ^a^	2.53 ± 1.34 ^b^	***
	E	1.00 ± 0.00 ^a^	1.13 ± 0.56 ^a^	1.27 ± 0.69 ^a^	2.07 ± 1.66 ^b^	***
		NS	NS	NS	NS	
Meat colour	C	7.00 ± 0.00 ^c^	6.32 ± 0.47 ^b^	4.68 ± 0.77 ^a^	4.07 ± 0.82 ^a,y^	***
	E	7.00 ± 0.00 ^c^	6.36 ± 0.49 ^b^	4.70 ± 0.46 ^a^	4.40 ± 0.67 ^a,x^	***
		NS	NS	NS	*	
Fat colour	C	7.00 ± 0.00 ^c^	6.32 ± 0.47 ^b^	4.68 ± 0.98 ^a^	4.50 ± 0.99 ^c,y^	***
	E	7.00 ± 0.00 ^c^	6.48 ± 0.51 ^b^	4.63 ± 0.49 ^a^	4.95 ± 0.75 ^a,x^	***
		NS	NS	NS	*	

Notes: 7-point scale (0 = not perceptible; 7 = maximum perceptible). Diet C: commercial feed, R: commercial feed supplemented with 1000 ppm of deodorized rosemary extract. Values within a row (^a,b,c^ effect of storage time) and in column (^x,y^ effect of dietary treatment) with different superscripts differ significantly at *p* < 0.05. The *p* values indicated by *, ** and *** for *p* < 0.05, *p* < 0.01 and *p* < 0.001, respectively. NS: not significant *p* ≥ 0.05.

**Table 6:** Effect of feed and storage on the sensory attributes in cooked meat of Chato Murciano pork stored under controlled conditions.

Attributes	Diet/Storage	Day 0	Day 14	*P*-Value
Meat odour	C	6.47 ± 0.70 ^b^	5.70 ± 0.92 ^a^	**
	E	6.20 ± 0.70	5.79 ± 0.86	NS
		NS	NS	
Rancid odour	C	1.21 ± 0.54 ^a^	1.75 ± 0.79 ^b^	*
	E	1.20 ± 0.41 ^a^	1.59 ± 0.78 ^b^	***
		NS	NS	
Acid odour	C	1.00 ± 0.00 ^a^	1.20 ± 0.41	*
	E	1.15 ± 0.34	1.26 ± 0.56	NS
		NS	NS	
Meat colour	C	6.42 ± 0.61	5.10 ± 0.72	NS
	E	6.40 ± 0.68 ^b^	5.84 ± 0.83 ^a^	*
		NS	NS	
Meat flavour	C	6.47 ± 0.45 ^b,y^	5.65 ± 1.18 ^a^	***
	E	6.25 ± 0.64 ^b,x^	5.83 ± 0.70 ^a^	**
		*	NS	
Rancid flavour	C	1.11 ± 0.32 ^a^	2.25 ± 1.07 ^b^	*
	E	1.15 ± 0.37 ^a^	2.00 ± 0.75 ^b^	***
		NS	NS	
Hardness	C	3.26 ± 0.45 ^a,x^	4.75 ± 0.72 ^b^	*
	E	3.60 ± 0.60 ^y^	4.00 ± 0.75	NS
		*	NS	
Juiciness	C	3.74 ± 0.99 ^b,y^	3.10 ± 0.79 ^a^	*
	E	4.05 ± 0.61 ^x^	3.68 ± 0.82	NS
		*	NS	

Notes: 7-point scale (0 = not perceptible; 7 = maximum perceptible). Diet C: commercial feed, R: commercial feed supplemented with 1000 ppm of deodorized rosemary extract. Values within a row (^a,b,c^ effect of storage time) and in column (^x,y^ effect of dietary treatment) with different superscripts differ significantly at *p* < 0.05. The *p* values indicated by *, ** and *** for *p* < 0.05, *p* < 0.01 and *p* < 0.001, respectively. NS: not significant *p* ≥ 0.05.

## Data Availability

Data available on request due to restrictions of privacy.
